# Management and outcomes of proteasome inhibitor associated chalazia and blepharitis: a case series

**DOI:** 10.1186/s12886-019-1118-x

**Published:** 2019-05-14

**Authors:** Bonnie A. Sklar, Kalla A. Gervasio, Siyang Leng, Arnab Ghosh, Ajai Chari, Albert Y. Wu

**Affiliations:** 1 Department of Ophthalmology, Icahn School of Medicine at Mount Sinai, One Gustave L. Levy Place Box 1183, New York, NY 10029 USA; 2 Wills Eye Hospital, 840 Walnut Street, Philadelphia, PA 19107 USA; 3 Department of Medicine, Hematology, and Oncology, Icahn School of Medicine at Mount Sinai, 1 Gustave L. Levy Pl, New York, NY 10029 USA; 4 Division of Hematology/Oncology, Columbia University Medical Center, 630 West 168th St., New York, NY 10032 USA; 5 Memorial Sloan Kettering Cancer Center, 1275 York Avenue, New York, NY 10065 USA; 6 Department of Ophthalmology, Byers Eye Institute, Stanford School of Medicine, 2452 Watson Court, Palo Alto, CA 94303 USA

**Keywords:** Chalazia, Blepharitis, Eyelid, Plasma cell disorder, Multiple myeloma, Proteasome inhibitor, Bortezomib, Chemotherapy

## Abstract

**Background:**

The purpose of this case series was to further characterize proteasome inhibitor associated chalazia and blepharitis, to investigate outcomes of different management strategies, and to propose a treatment algorithm for eyelid complications in this patient population.

**Methods:**

This retrospective case series included sixteen patients found to have chalazia and/or blepharitis while receiving proteasome inhibitors for plasma cell disorders at Mount Sinai Hospital in New York, NY from January 2010 through January 2017. Main outcomes were complete resolution of eyelid complications and time to resolution. Student’s t-test was used to compare average values and Fisher’s exact test was used to compare proportions.

**Results:**

Fourteen patients had chalazia and 10 had blepharitis. Chalazia averaged 5.4 mm, and 11 patients with chalazia experienced two or more lesions. Median follow-up time was 17 months. Average time from bortezomib exposure to onset of first eyelid complication was 3.4 months. Chalazia episodes were more likely to completely resolve than blepharitis episodes (*p* = 0.03). Ocular therapy alone was trialed for an average of 1.8 months before proceeding to bortezomib omission. Average time to eyelid complication resolution using ocular therapy alone was 1.8 months versus 3.1 months after bortezomib omission. In this series, the combination of ocular therapy and bortezomib omission led to complete resolution of eyelid complications more often than ocular therapy alone.

**Conclusion:**

Proteasome inhibitor associated eyelid complications were identified in sixteen patients with plasma cell disorders. Eyelid complications may be treated with a 2-month trial of conservative ocular therapies alone, followed by continuation of ocular therapy in combination with bortezomib omission if eyelid signs persist.

## Background

Proteasome inhibitors, specifically bortezomib (Velcade), have been associated with ocular complications in patients with plasma cell disorders [1–5]. Bortezomib is approved for treatment of multiple myeloma, relapsed mantle cell lymphoma, and additional hematologic malignancies [6, 7]. Common side effects include peripheral neuropathy, thrombocytopenia, neutropenia, gastrointestinal toxicities, herpes zoster reactivation, and other infections [6, 8, 9]. Chemotherapy-associated ocular complications and their management have been less well-characterized.

Previous reports have linked bortezomib with development of severe bilateral blepharitis, a chronic inflammatory eyelid process, and formation of chalazia, lipogranulomatous lesions that develop

secondary to Meibomian gland dysfunction [1–5, 10]. Bortezomib-associated chalazia may be refractory to conservative treatments, instead requiring incision and curettage [5].

In the largest case series to date, we identified sixteen patients who presented with eyelid complications following systemic proteasome inhibitor therapy for plasma cell disorders. Eyelid complications included a single chalazion, multiple chalazia affecting multiple eyelids, and/or blepharitis. The purpose of this case series was to further characterize proteasome inhibitor associated chalazia and blepharitis, to investigate outcomes of different management strategies, and to propose a treatment algorithm for eyelid complications in this patient population.

## Methods

This retrospective case series was approved by the ethics committee at Mount Sinai Hospital and adhered to HIPAA regulations and the Declaration of Helsinki. A waiver of authorization for the release of protected health information for research purposes was granted by the Mount Sinai Institutional Review Board. As this was a retrospective study with de-identified data, informed consent was not required.

Seventeen patients on proteasome inhibitors for plasma cell disorders who presented with comorbid eyelid complications were identified through the Multiple Myeloma Program at Mount Sinai Hospital in New York, NY from January 2010 to January 2017. All patients were subsequently referred to Mount Sinai’s ophthalmology clinic for further work-up. Patients were included if they had a plasma cell disorder diagnosis, had been treated with a proteasome inhibitor, and were subsequently found to have blepharitis and/or chalazia. One patient was excluded after further chart review because neither blepharitis nor chalazia were found on ophthalmologic examination. Patients were seen by one of four physicians in the Multiple Myeloma program for their cancer diagnosis and one ophthalmic plastic surgeon for their eyelid complications.

Retrospective chart review noted patient demographics, cancer diagnosis, chemotherapy regimen, ocular diagnoses and management. The horizontal width of each chalazion was also measured prior to treatment. Ocular treatments included hot compresses, topical antibiotic and/or steroid drops and ointment, systemic antibiotics, oral steroids, incision and curettage, or observation. Ocular therapy choice was up to the ophthalmologist’s discretion.

Ocular complications are reported on a patient-by-patient basis, as well as by “episodes.” The date that a new complication begins is considered the start of an episode. Patients may have more than one lesion during a single episode (i.e., multiple chalazia), and patients may have multiple episodes throughout their history (i.e., a new set of chalazia emerges at a later date). Main outcomes noted were complete resolution of chalazia and/or blepharitis episodes and time to resolution. Statistical analysis was performed using SPSS (Release 24.0.0.0, PASW Statistics 24, Polar Engineering and Consulting). Student’s t-test was used to compare average values and Fisher’s exact test was used to compare proportions. The cutoff for *p*-value significance was p less than 0.05 (two-tailed) at a 95% confidence interval.

## Results

Sixteen patients treated with proteasome inhibitors for plasma cell disorders were found to have chemotherapy-associated eyelid complications. Patient demographics and chemotherapy regimens are summarized in Table 1. Four (25%) patients developed their first ocular complication during the first cycle of proteasome inhibitor therapy, while the remaining 12 (75%) had received multiple cycles. Patients were followed for a median duration of 17 months.

**Table 1:** Demographics and chemotherapy regimens of sixteen patients with plasma cell disorders and proteasome inhibitor-associated eyelid complications

Patients (n)	16
Average age (range)	62 (40–82)
Male (n)	5 (31%)
Female (n)	11 (69%)
Multiple myeloma isotype (n)	
IgG^a^	10 (71%)
IgA^b^	3 (21%)
Light Chain	1 (7%)
AL^c^ amyloidosis with MGUS^d^ (n)	2 (12.5%)
AL amyloid (n)	
Yes	4 (25%)
No	10 (62.5%)
Not tested	2 (12.5%)
ISS^v^ at diagnosis (median)	1
Complete lines of therapy prior to ocular complication (n)	
0	12 (75%)
1	1 (7%)
2	2 (12.5%)
3	1 (7%)
Bortezomib exposure (n)	
Naïve	12 (75%)
Previous exposure	4 (25%)
Chemotherapy regimen at initial chalazia episode (n)	
Bortezomib, Lenalidomide, Dexamethasone	9 (64%)
Bortezomib, Cyclophosphamide, Dexamethasone	3 (21%)
Bortezomib, Dexamethasone	1 (7%)
Carfilzomib, Pomalidomide, Dexamethasone	1 (7%)
Chemotherapy at initial blepharitis episode (n)	
Bortezomib, Lenalidomide, Dexamethasone	6 (60%)
Bortezomib, Cyclophosphamide, Dexamethasone	2 (20%)
Bortezomib, Cyclophosphamide, Pomalidomide, Thalidomide	1 (10%)
Carfilzomib, Cyclophosphamide, Dexamethasone	1 (10%)
Concurrent steroid use at time of initial complication (n)	16 (100%)
Average time to onset of first eyelid complication (range)	103 days (6–258)

^a^IgG = immunoglobulin G

^b^IgA = immunoglobulin A

^c^AL amyloidosis = amyloid light-chain amyloidosis

^d^ISS = International Staging System score for multiple myeloma

Ocular complications are summarized in Table 2. Fourteen (87.5%) patients had chalazia (23 episodes) and 10 (62%) patients had blepharitis (11 episodes). Eight (50%) patients had both chalazia and blepharitis. No patients had a history of preexisting seborrheic dermatitis, rosacea, or prior ocular infections. Of the fourteen patients with chalazia, eleven (79%) experienced two or more concurrent lesions. Female patients were more likely to experience two or more concurrent lesions per episode than male patients (*p* = 0.01), while patients with amyloid were less likely than non-amyloid patients to have two or more concurrent lesions per episode (*p* = 0.04). Slit lamp examinations were available for ten patients with chalazia, with lesions measuring an average of 5.4 mm (range 2–15 mm) prior to any ocular therapy.

**Table 2:** Ocular complications secondary to proteasome inhibitor therapy for sixteen patients with plasma cell disorders

Ocular complications by diagnosis (*n* = 24):	
Chalazion	14 (87.5%)
Blepharitis	10 (62%)
Ocular complications by episode (*n* = 34):	
Chalazion	23 (68%)
Blepharitis	11 (32%)
Ocular complications by patient (*n* = 16):	
Chalazion only	6 (37.5%)
Blepharitis only	2 (12.5%)
Chalazion + blepharitis	8 (50%)
Chemotherapy-associated eyelid complication characteristics:	
Patients with chalazia (n)	14
Episodes of chalazia (n)	23
Patients with 2+ chalazia at single episode (n)	11 (79%)
Average chalazia at most severe episode (range)	2.7 (1–6)
Average chalazion size in mm (range)	5.4 (2–5)
Average total affected eyelids (range)	2.4 (1–4)
Total chalazia in cohort (n)	46
Upper eyelid chalazia	32 (70%)
Lower eyelid chalazia	14 (30%)
Laterality	
Bilateral chalazia (episodes)	12 (52%)
Unilateral chalazia (episodes)	11 (48%)
Bilateral blepharitis (episodes)	9 (82%)
Unilateral blepharitis (episodes)	1 (9%)
Blepharitis laterality unknown (episodes)	1 (9%)

All sixteen patients received systemic proteasome inhibitor therapy, in combination with other chemotherapy agents, prior to presenting with eyelid complications. Fifteen (94%) patients received a regimen including bortezomib at their initial eyelid complication; the sixteenth patient received carfilzomib, a newer generation proteasome inhibitor. Two eyelid complication episodes occurred while patients were on carfilzomib. One chalazia episode occurred in a patient who had completed prior therapy lines with bortezomib 7 months before initiating a new line of therapy with carfilzomib. One blepharitis episode occurred in a patient for whom bortezomib had been discontinued and immediately switched to carfilzomib. Blepharitis occurred 3.5 months after carfilzomib initiation and 6.5 months after bortezomib cessation.

**Fig. 1:**
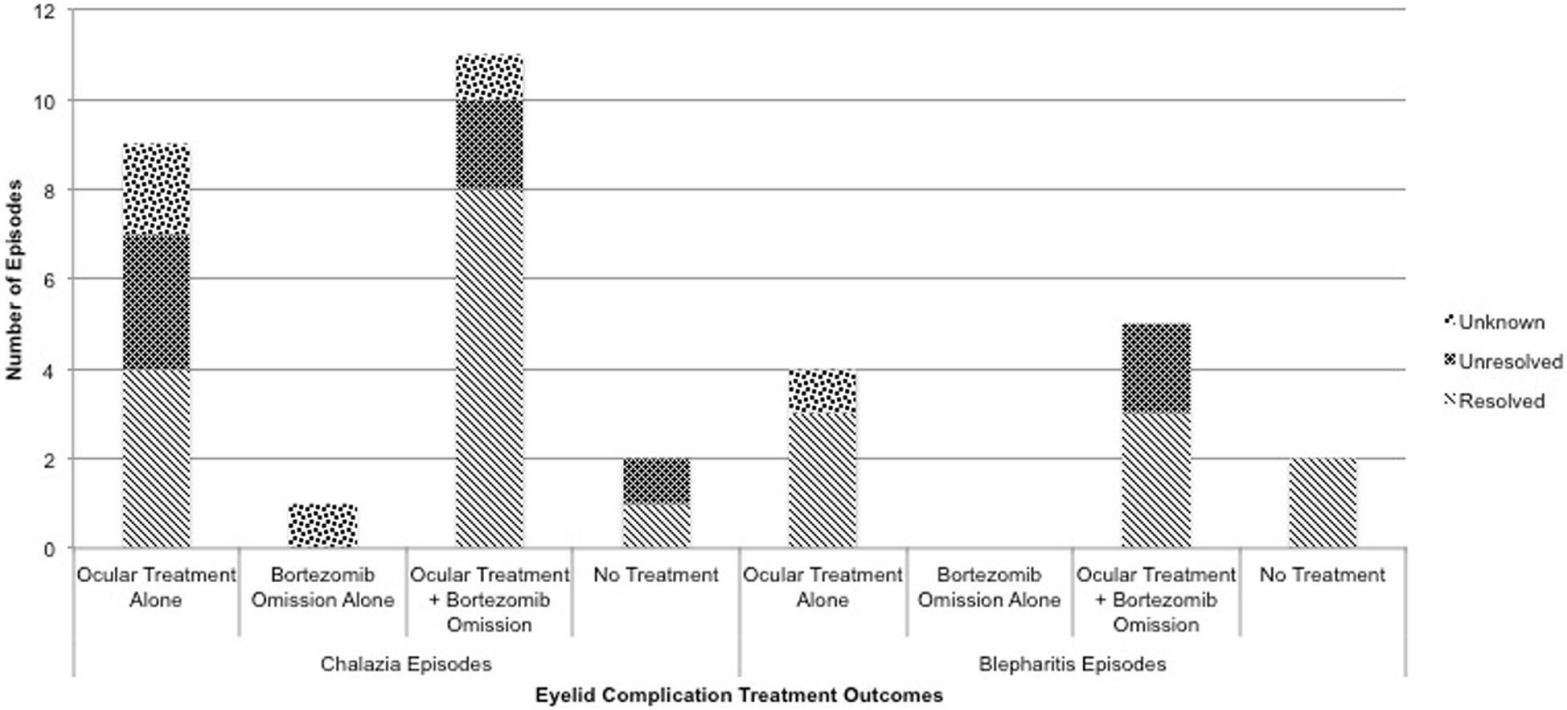
Management and outcomes of eyelid complications in sixteen patients with plasma cell disorders on proteasome inhibitors. A stacked bar graph demonstrating the number of episodes of chalazia and blepharitis that resolved or persisted based on treatment with ocular therapy alone, bortezomib omission alone, a combination of the two, or no treatment/observation

**Fig. 2:**
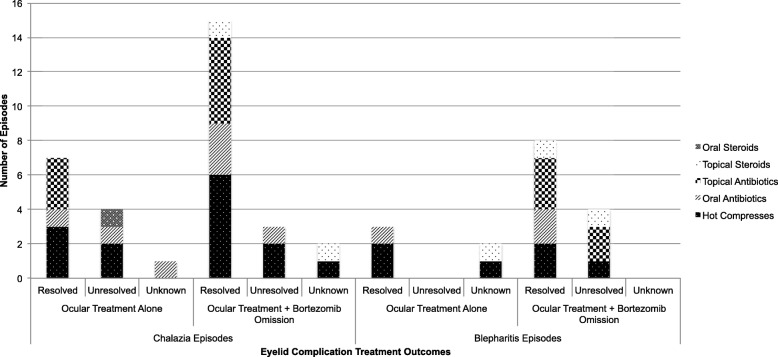
Outcomes of specific ocular treatment options used for eyelid complications in sixteen patients with plasma cell disorders on proteasome inhibitor therapy. A stacked bar graph demonstrating the number of episodes of chalazia and blepharitis that resolved or persisted based on specific ocular therapies such as hot compresses, topical antibiotics and/or steroids, oral antibiotics, or oral steroids. Topical antibiotic and/or steroid drops and ointments used included tobramycin, tobramycin/dexamethasone, azithromycin, erythromycin, bacitracin, loteprednol, and dexamethasone. Systemic antibiotics used included doxycycline, cephalexin, gentamicin, and ciprofloxacin. Oral steroids used included methylprednisolone

Of 34 eyelid complication episodes, outcomes are known in 29 (Fig. 1). The most common ocular treatments for patients included warm compresses (18 episodes), systemic antibiotics (9 episodes), topical antibiotic eye drops and/or ointment (12 episodes), and topical steroid drops and/or ointment (4 episodes). Due to the retrospective nature of this case series, treatment regimens and patient education were not standardized. Furthermore, patient compliance rates may have varied. Refer to Fig. 2 for outcomes of specific ocular therapies. During episodes in which bortezomib was discontinued, 7/17 times it was solely withheld (3 episodes resolved), while 8/17 times it was withheld and switched to ixazomib or carfilzomib (6 episodes resolved). There were 19 chalazia episodes with known post-treatment outcomes, and 4 episodes of unknown outcome that were excluded. Of nine chalazia episodes treated with ocular-directed therapy alone, 4 (44%) completely resolved. Of ten chalazia episodes treated with ocular therapy and bortezomib omission, 8 (80%) completely resolved. There were 10 blepharitis episodes with known post-treatment outcomes, and 1 episode of unknown outcome that was excluded. Three (100%) episodes treated with ocular therapy alone resolved completely, while 3 (60%) of the 5 episodes with ocular treatment and bortezomib omission completely resolved.

In patients treated with ocular therapies alone, the average time to eyelid complication resolution was 55 days (1.8 months). In patients treated with ocular therapies alone whose eyelid complications did not resolve, the average amount of time that conservative therapy was trialed was 53 days (1.8 months), before proceeding to bortezomib omission. In patients in which bortezomib was subsequently omitted, the average time from omission to resolution of eyelid complications was 93 days (3.1 months). Given the variability in ocular therapies utilized, these results should be taken with caution until more standardized studies have been completed.

Complete resolution of eyelid complication episodes did not depend on specific treatment approach, when considering ocular therapy alone or in combination with bortezomib omission, or observation with no intervention (*p* = 0.72). Episodes of chalazia were more likely to completely resolve than episodes of blepharitis (*p* = 0.03). There was no difference in complete resolution of eyelid complication episodes based on patient gender (*p* = 0.21), age (*p* = 0.58), multiple myeloma isotype (*p* = 0.47), presence of amyloid (*p* = 0.39), multiple myeloma international staging system score (*p* = 0.52), or chemotherapy cycle at which the eyelid syndrome was diagnosed (*p* = 0.09). There was also no difference in complete resolution of episodes based on whether or not bortezomib was solely omitted versus switched to an alternate proteasome inhibitor (*p* = 0.59).

Only one patient underwent bortezomib rechallenge. This patient had originally experienced an episode of 3 concurrent chalazia 3 months into bortezomib therapy, which resolved upon bortezomib discontinuation in combination with ocular therapy (hot compress, doxycycline, and erythromycin ointment). Chemotherapy was switched to carfilzomib for 5 months. Bortezomib rechallenge occurred during three separate time periods over the course of a year. Chalazia or blepharitis never redeveloped. No other patients underwent rechallenge, as they were either switched to carfilzomib or ixazomib, had bortezomib discontinued permanently, or had resolution of eyelid complications with ocular therapy alone.

## Discussion

This retrospective case series contributes to the growing literature on the association between proteasome inhibitors and chalazia and blepharitis [1–5]. To our knowledge, 28 cases of bortezomib-associated chalazia and blepharitis have been reported [3, 5]. Most recently, the association between bortezomib and chalazia was classified as a “possible” adverse drug reaction (ADR) based on the World Health Organization’s definition of ADRs and an analysis of both case reports in the literature as well as reports submitted to the National Registry of Drug-Induced Ocular Side Effects [3].

In the largest case series to date, we identified 16 patients on proteasome inhibitors for plasma cell disorders who presented with chemotherapy-associated eyelid complications. In accordance with prior case reports, our patients had onset of their initial eyelid complication(s) at an average of 3.4 months [1, 3]. In our series, 44% of chalazia episodes and 100% of blepharitis episodes treated with ocular therapy alone completely resolved. For episodes in which ocular therapy was used in combination with bortezomib omission, 80% of chalazia episodes and 60% of blepharitis episodes resolved. The difference in time to resolution using ocular therapy alone versus ocular therapy combined with bortezomib omission approached statistical significance at *p* = 0.05.

Cases in which chalazia and/or blepharitis persisted after bortezomib omission were not unexpected, as patients were followed for a median of only 17 months, and previous case reports have shown that eyelid complications may last for many months after bortezomib cessation [1]. It is therefore possible that unresolved episodes may have resolved at a point beyond follow-up time observed. Overall, episodes of chalazia were significantly more likely to completely resolve than blepharitis episodes. Of note, when bortezomib was omitted, blepharitis episodes took on average a longer period of time (117 days) than chalazia episodes to completely resolve (74 days), with no significant difference between them. No patients in our study received a prolonged course of oral doxycycline for persistent chalazia or blepharitis after bortezomib omission, which has previously been used successfully in 2 cases [5].

The specific pathogenesis of blepharitis and chalazia secondary to proteasome inhibitor therapy is unknown, but is postulated to be related to inflammation. Bortezomib is a proteasome inhibitor that inhibits the ubiquitin proteasome pathway leading to the accumulation of pro-apoptotic molecules and thereby apoptosis of neoplastic cells. Accumulation of degraded proteins in the meibomian glands may lead to eyelid complications. Bortezomib also influences critical inflammatory pathways including NF-kB, JAK/STAT, and MAP kinase, promoting release of pro-inflammatory cytokines [11–13]. Interference of these pathways in the eyelids may result in inflammatory flares causing blepharitis and/or chalazia [14]. All patients in this series were receiving dexamethasone as part of their chemotherapy regimen. Interestingly, in one patient, blepharitis had resolved while on 40 mg of intravenous (IV) dexamethasone, but then flared when the dose was decreased to 10 mg IV. Recurrence and flare of blepharitis at a lower steroid dose supports the idea that the pathogenesis of eyelid complications secondary to proteasome inhibitors is more likely an inflammatory mediated process than an infectious one. None of our patients had their eyelid lesions cultured, so infectious causes could not be definitively ruled out.

If pathogenesis is indeed related to inflammation, this may explain why anti-inflammatory ocular treatments in our study such as systemic antibiotics like doxycycline or topical antibiotics like tobramycin were effective in resolving chalazia and/or blepharitis. Ocular treatments in our study ranged from warm compresses to topical antibiotic and/or steroid drops and ointment to bortezomib discontinuation. In this series, a combination of bortezomib discontinuation and ocular treatments was effective more often than ocular treatments alone. Further studies are needed to elucidate whether specific systemic antibiotics versus topical antibiotic and/orf steroids are more effective than others in treating proteasome-inhibitor associated eyelid complications.

**Fig. 3:**
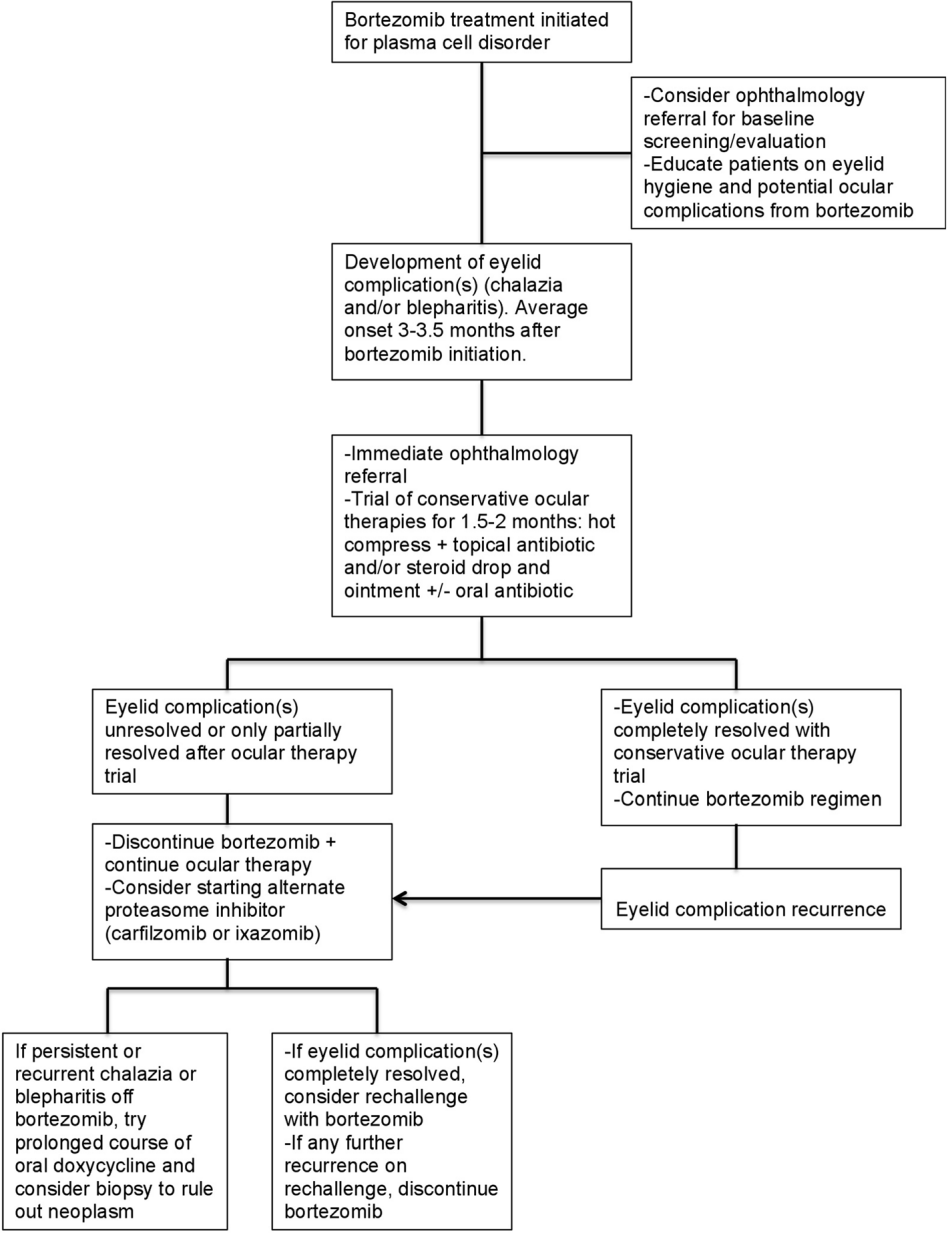
Treatment algorithm. A proposed management sequence for patients with plasma cell disorders and proteasome inhibitor-associated eyelid complications

Based on our experience with the largest series of proteasome inhibitor-associated chalazia and blepharitis patients to date, we propose the following treatment algorithm (Fig. 3). Upon bortezomib initiation, oncologists may consider an optional ophthalmology referral for a baseline eye exam, patient education on ocular complications of bortezomib, and instruction on preventive eyelid hygiene. Patients can be informed that eye complications tend to occur within 3–3.5 months of bortezomib initiation. Without knowing the specific incidence and prevalence of bortezomib-associated eyelid complications, this baseline eye screening is up to the oncologist’s discretion and while not imperative, it is encouraged. Upon initiation of the first eyelid complication, immediate ophthalmology referral should be made and subsequent 1.5–2 month trial of conservative ocular therapies employed.

Ocular therapy choice is up to the ophthalmologist’s discretion, but we propose using hot compresses in combination with at least 1 topical antibiotic and/or steroid drop, and possible oral antibiotics as well. A prospective, randomized study conducted by the senior author of this manuscript found that hot compresses alone or in combination with tobramycin or tobramycin/dexamethasone drops and ointment are all effective first-line treatment options for conventional chalazia [15]. In our study, no specific combination of conservative therapies could be evaluated as more effective in this population due to small sample size. If eyelid complications persist after a 1.5–2 month trial of ocular therapy alone, proceed to bortezomib omission plus continuation of ocular therapy, and consider possible switch to an alternate proteasome inhibitor (carfilzomib or ixazomib) depending on the patient’s oncologic status. If eyelid signs persist after bortezomib omission, consider specific addition of oral doxycycline, which has been shown to resolve persistent blepharitis after bortezomib cessation in prior studies [5]. If eyelid complications resolve after bortezomib omission, bortezomib may be rechallenged once, based on the patient’s oncologic status and willingness depending on prior severity of eyelid complications. However, if eyelid complications recur upon bortezomib rechallenge, the drug should be discontinued again.

Limitations of our study include small sample size and retrospective design. Larger prospective studies are needed to determine the true incidence and prevalence of proteasome inhibitor-associated eyelid complications, as well as the most effective ocular treatments. More data is needed regarding specific time periods when patients can be rechallenged with bortezomib, or if they may be rechallenged at all. In our study, only one patient underwent bortezomib rechallenge, making interpretation of this clinical situation more challenging. Our study could have been strengthened by using a standardized method to grade severity of each chalazion and blepharitis episode in order to see how severity affected management and outcomes. However, due to the retrospective nature of this study, no specific severity grading systems were employed.

## Conclusions

Proposed management for proteasome inhibitor-associated eyelid complications includes a 2 month trial of conservative ocular therapies alone, followed by continuation of ocular therapy in combination with bortezomib omission if eyelid lesions persist. When bortezomib is omitted, possible switch to an alternate proteasome inhibitor such as carfilzomib or ixazomib may be considered. We find that the combination of ocular therapy and bortezomib omission led to complete resolution of eyelid complications more often than ocular therapy alone in this series. Awareness and prompt management of these eyelid complications may help improve quality of life for patients receiving proteasome inhibitors for plasma cell disorders.

## Abbreviations

ADR: Adverse Drug Reaction
AL Amyloidosis: Amyloid light-chain amyloidosis
IgA: Immunoglobulin A
IgG: Immunoglobulin G
ISS: International Staging System score for multiple myeloma
MGUS: Monoclonal Gammopathy of Undetermined Significance
